# Gut–Liver Axis Dysfunction in Alcohol-Associated Liver Disease and the Potential Role of Sheep Yogurt: A Scoping Review and Mechanistic Framework

**DOI:** 10.3390/nu18152549

**Published:** 2026-08-04

**Authors:** Yunfeng Wu, Yulong Zhao, Wenna Yao, Yanyan Yang, Hui Bai, Siqin Bao, Xihe Li, Yongli Song

**Affiliations:** 1 Research Center for Animal Genetic Resources of Mongolia Plateau, College of Life Sciences, Inner Mongolia University, Hohhot 010020, China; 19803425516@163.com (Y.W.); z1369609005@outlook.com (Y.Z.); yaowenna024@163.com (W.Y.); baosq@imu.edu.cn (S.B.); 2 The State Key Laboratory of Reproductive Regulation and Breeding of Grassland Livestock, College of Life Sciences, Inner Mongolia University, Hohhot 010020, China; 3 Inner Mongolia Academy of Agricultural & Animal Husbandry Sciences, Huhhot 010000, China; swallow_0088@163.com (Y.Y.); bbbbbhhhww@163.com (H.B.); 4 Inner Mongolia Saikexing Institute of Breeding and Reproductive Biotechnology in Domestic Animal, Hohhot 011517, China

**Keywords:** alcohol-associated liver disease, sheep yogurt, gut-liver axis, fermented dairy, intestinal barrier, microbiome, mycobiome, ovine milk

## Abstract

**Background/Objectives**: Alcohol-associated liver disease (ALD) is driven by gut-liver axis dysfunction, including intestinal barrier disruption, dysbiosis, microbial translocation, inflammation, metabolic dysfunction, and malnutrition. Fermented dairy foods may modulate several of these domains, yet whether sheep yogurt, as an intact fermented dairy matrix, is relevant in ALD is unknown. This scoping review mapped evidence relevant to sheep yogurt, ALD, and gut-liver axis biology. **Methods**: A PRISMA-ScR-guided scoping review searched PubMed/MEDLINE, Web of Science, Scopus, and Google Scholar from January 2006 to February 2026. Eligible sources were charted using a prespecified framework classifying evidence as direct, indirect, or mechanistic inference. Mapped domains included ALD pathophysiology; intestinal barrier integrity; bacterial and fungal microbial ecology; bile acid and tryptophan-aryl hydrocarbon receptor signaling; nutritional vulnerability; fermented dairy interventions; and ovine dairy-matrix characteristics. **Results**: Of 1388 records identified, 121 sources were included after duplication and screening. No eligible study directly tested sheep yogurt or a defined sheep yogurt preparation in ALD-relevant experimental or clinical settings. Indirect evidence supported the relevance of gut-liver axis dysfunction to ALD and indicated that selected fermented dairy products, probiotics, postbiotics, and microbial preparations may influence intestinal permeability, inflammatory signaling, microbial ecology, oxidative stress, and liver-injury outcomes. Compositional data supported sheep yogurt as a distinct food matrix. However, findings from isolated components, probiotic-only interventions, and non-ALD models could not be interpreted as evidence of sheep yogurt efficacy in ALD. **Conclusions**: The current literature supports a hypothesis-driven research framework rather than any therapeutic claim for sheep yogurt in ALD. Any potential benefit of sheep yogurt in ALD remains hypothetical and cannot support clinical or dietary recommendations until validated experimentally. Future direct, comparator-controlled studies of intact sheep yogurt should assess liver injury, barrier integrity, microbial translocation, relevant metabolites, and nutrition-related outcomes.

## 1. Introduction

Alcohol-associated liver disease (ALD) represents a major clinical and public health burden and includes a spectrum of liver injury ranging from steatosis and steatohepatitis to fibrosis, cirrhosis, alcohol-associated hepatitis, and hepatocellular carcinoma [1,2]. Disease progression is influenced not only by alcohol exposure itself, but also by nutritional status, immune activation, metabolic comorbidity, infection risk, and gut-derived inflammatory signals [3,4]. Because many of these factors are potentially modifiable, nutritional and food-based approaches have attracted increasing interest as adjunctive strategies for improving gut–liver axis resilience in ALD [5].

The gut-liver axis provides a focused rationale for nutritional interventions in ALD. Alcohol disrupts mucus defense and tight junctions, increases intestinal permeability and microbial translocation, and thereby promotes hepatic inflammatory signaling [6,7,8,9]. ALD is also associated with bacterial and fungal dysbiosis, including altered microbial ecology and Candida-related immune activation [10,11,12,13]. Together, these disturbances perturb barrier function, bacterial and fungal ecology, microbial translocation, and host inflammatory and metabolic responses as relevant outcome domains for evaluating candidate nutritional matrices.

Fermented dairy products are biologically relevant in this context because they may deliver viable microorganisms, non-viable microbial components, fermentation-derived metabolites, proteins, peptides, lipids, minerals, and other matrix-associated compounds [14,15]. Studies of yogurt, fermented milk, kefir, probiotics, postbiotics, and lactic acid bacteria have reported effects on intestinal permeability, microbial composition, oxidative stress, inflammatory signaling, and liver-injury-related outcomes in selected experimental or clinical contexts [16,17,18]. However, an important unresolved issue is whether evidence from isolated probiotics, postbiotics, purified peptides, or non-ovine fermented dairy products can be transferred to an intact sheep yogurt matrix. These intervention types differ in composition, viability, digestion behavior, nutrient delivery, and food-matrix structure; therefore, they should not be treated as interchangeable [19].

Sheep yogurt may represent a distinct fermented dairy matrix. Ovine milk generally differs from bovine and caprine milk in total solids, protein content, lipid composition, mineral concentration, casein and whey protein profile, and physicochemical properties [20,21]. Fermentation may further modify acidity, texture, protein structure, peptide release, microbial viability, and metabolite availability [22]. These characteristics provide a rationale for examining sheep yogurt as a whole-food matrix rather than as a generic yogurt, probiotic carrier, or source of isolated dairy components. Nevertheless, compositional distinctiveness alone does not establish efficacy in ALD, and evidence from non-ALD models or isolated components should be interpreted cautiously.

Several pathways may be informative for future testing, including barrier regulation, bacterial and fungal ecology, bile acid signaling, tryptophan-AhR signaling, oxidative and inflammatory responses, and nutritional vulnerability [23,24,25,26,27]. However, the evidence derives from heterogeneous sources, including ALD mechanisms, non-ovine fermented dairy products, isolated probiotics, postbiotics and their components, ovine-milk composition studies, digestion studies, and non-ALD models. Accordingly, this evidence cannot be interpreted as direct support for sheep yogurt efficacy in ALD.

Therefore, this PRISMA-ScR-guided scoping review maps evidence relevant to sheep yogurt, ALD, and gut-liver axis biology. Distinct from previous reviews of fermented dairy products, probiotics, or the gut-liver axis, this review treats sheep yogurt as an intact ovine-derived fermented dairy matrix, explicitly separates direct evidence from indirect and mechanistic evidence, and defines the comparator-controlled designs required for matrix-level attribution. Specifically, the review aimed to determine whether sheep yogurt or defined sheep yogurt preparations have been directly tested in ALD-relevant experimental or clinical systems; synthesize adjacent evidence from ALD, fermented dairy, probiotic, postbiotic, and ovine dairy studies; identify priority pathways and outcome domains for future research; and define evidence boundaries that limit causal or therapeutic interpretation. Thus, the review establishes a hypothesis-driven research framework rather than a therapeutic for sheep yogurt in ALD.

## 2. Materials and Methods

This scoping review was conducted according to the PRISMA extension for scoping reviews (PRISMA-ScR), using a structured and reproducible search strategy aimed at integrating nutritional, microbiological, mechanistic, and experimental evidence relevant to sheep yogurt, alcohol-associated liver disease (ALD), and gut-liver axis biology. The review was designed to map the available evidence and define evidence boundaries, rather than to estimate a pooled intervention effect. Given the expected heterogeneity of the literature, including clinical ALD studies, ethanol-induced liver injury models, fermented dairy interventions, probiotic and postbiotic studies, ovine dairy composition studies, and pathway-based mechanistic evidence, a narrative evidence-mapping approach was applied. All figures in this manuscript were prepared using Adobe Illustrator (version 29.6.1, 64-bit).

### 2.1. Information Sources and Search Strategy

Literature searches were conducted across PubMed/MEDLINE, Scopus, Web of Science Core Collection, and Google Scholar. Searches covered publications from 1 January 2006 to 12 February 2026. This time window was selected to capture the earliest relevant study identified during the searches while incorporating recent evidence relevant to ALD pathophysiology, fermented dairy biology, ovine milk composition, and gut-liver axis mechanisms. The complete database-specific search strategies, search dates, and date limits are provided in Supplementary Table S1.

The search strategy was developed around complementary evidence domains. The first domain focused on ALD and ethanol-related liver injury, including alcohol-associated liver disease, alcohol-related liver disease, alcoholic liver disease, alcoholic hepatitis, ethanol-induced liver injury, and alcohol-induced liver injury. The second domain focused on gut–liver axis mechanisms, including intestinal barrier dysfunction, intestinal permeability, tight junctions, mucus defense, microbial translocation, lipopolysaccharide, endotoxemia, microbiota, microbiome, mycobiome, Candida, and fungal translocation. The third domain focused on metabolic, immune, and nutritional pathways relevant to ALD, including bile acid metabolism, farnesoid X receptor, TGR5, FGF15/19, tryptophan metabolism, indole derivatives, aryl hydrocarbon receptor signaling, zinc status, malnutrition, and sarcopenia. The fourth domain focused on fermented dairy products, probiotics, postbiotics, lactic acid bacteria, yogurt, fermented milk, kefir, and microbial preparations. The fifth domain focused on ovine dairy and sheep yogurt matrix characteristics, including sheep milk, ewe milk, ovine milk, sheep yogurt, ovine yogurt, fermentation, digestion, dairy peptides, minerals, medium-chain fatty acids, and food-matrix effects. The structured search strategy, representative search terms, information sources, and purpose of each search block are summarized in Table 1, while the complete database-specific search strings are provided in Supplementary Table S1.

Relevant studies were identified using Medical Subject Headings, keyword combinations, and Boolean operators adapted to the syntax of each database. Search terms related to ALD and ethanol-induced liver injury were combined with terms related to gut–liver axis dysfunction, intestinal barrier integrity, microbial ecology, fermented dairy products, probiotics, postbiotics, ovine milk, sheep yogurt, and mechanistic pathways. Google Scholar was used as a supplementary source for targeted searches, citation tracking, and identification of additional relevant studies. The bibliographic references of relevant primary studies and reviews were also screened manually to identify further sources.

### 2.2. Eligibility Criteria and Evidence Classification

Sources were considered eligible if they contributed evidence relevant to at least one predefined domain, including ALD or ethanol-induced liver injury; intestinal barrier integrity; bacterial microbiome or fungal mycobiome alterations; microbial or fungal translocation; bile acid metabolism; tryptophan-aryl hydrocarbon receptor signaling; nutritional vulnerability; fermented dairy, probiotic, or postbiotic interventions; ovine milk composition; sheep yogurt matrix characteristics; or food-matrix and digestion-related mechanisms. Direct evidence was defined as studies testing sheep yogurt or a defined sheep yogurt preparation in ALD, ethanol-induced liver injury, alcohol-associated hepatitis, or ALD-relevant gut-liver axis models. Indirect evidence included studies addressing ALD pathophysiology, fermented dairy products, probiotics, postbiotics, ovine milk composition, or sheep yogurt-related matrix features without directly testing sheep yogurt in ALD. Mechanistic inference included pathway, component, model-system, or comparator evidence that informed the biological plausibility but did not establish sheep yogurt efficacy.

### 2.3. Study Selection and Data Charting

All retrieved records were imported into Zotero version 9.0.6 (64-bit) for reference management and duplicate removal before screening. Records were screened initially by title and abstract, followed by full-text assessment when necessary. Sources were excluded if they were unrelated to ALD, gut-liver axis biology, fermented dairy, ovine dairy, sheep yogurt, or the predefined mechanistic domains; if they lacked sufficient relevance to the sheep yogurt matrix framework; or if they could not support classification as direct evidence, indirect evidence, or mechanistic inference.

For each eligible source, data were charted using a structured evidence-mapping framework. Extracted information included source type, disease or model context, intervention or exposure, dairy matrix or microbial component, gut-liver axis domain, main mechanistic endpoint, relevance to ALD, evidence category, and transferability boundary. The charted data were used to distinguish direct evidence from indirect evidence and mechanistic inference. The evidence-classification framework used for source charting and synthesis is shown in Table 2.

### 2.4. Evidence Synthesis

**Figure 1:**
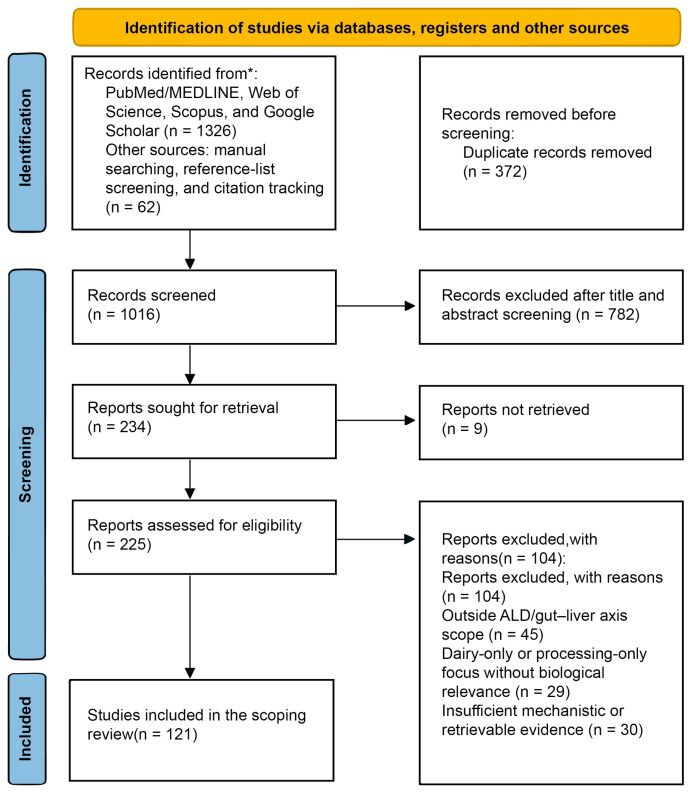
PRISMA-ScR flow diagram of study identification, screening, full-text assessment, and inclusion. The numbers of records at each stage and the reasons for full-text exclusion are presented in the flow diagram. * indicates statistical significance compared to the control group (* *p* < 0.05).

Evidence was synthesized narratively according to the predefined evidence domains and evidence-classification framework. Sources were grouped into direct evidence, indirect evidence, or mechanistic inference according to their intervention, disease context, model system, and transferability to sheep yogurt in ALD. Findings from isolated probiotics, postbiotics, dairy-derived components, non-ovine fermented dairy products, and non-ALD models were interpreted as adjacent evidence only and were not considered sufficient to support therapeutic claims for sheep yogurt in ALD. The source-selection process was documented using a PRISMA-ScR flow diagram (Figure 1). A total of 1388 records were identified from database searches and other sources; 372 duplicates were removed, leaving 1016 records for title and abstract screening. Of these, 782 records were excluded, and 234 reports were sought for retrieval; 9 reports could not be retrieved. A total of 225 full-text reports were assessed for eligibility, of which 104 were excluded because they were outside the ALD/gut–liver axis scope (*n* = 45), had a dairy-only or processing-only focus without biological relevance (*n* = 29), or provided insufficient mechanistic or retrievable evidence (*n* = 30). Overall, 121 sources were included in the scoping review.

## 3. ALD as a Gut-Liver Axis Disorder: Core Pathogenic Mechanisms

ALD diagnostics, staging, transplantation, and pharmacotherapy are outside the scope of this review. This chapter summarizes ALD pathophysiology only to the extent needed to support the sheep yogurt matrix framework developed below.

### 3.1. Bacterial Dysbiosis, Barrier Failure, and Microbial Translocation

Chronic ethanol exposure produces reproducible shifts in the intestinal bacterial community in human ALD and rodent models, including reductions in commensals associated with short-chain fatty acid (SCFA) production and relative expansion of opportunistic taxa [11,28,29]. Sarin et al. [30] reviewed the microbiome as a therapeutic target in alcohol-related liver disease, while Mendes and Schnabl [29] described the transition from intestinal dysbiosis to ALD. These changes co-occur with altered luminal pH, organic acid pools, and bile acid composition, defining a dysbiotic ecology that is unlikely to be corrected by single-agent strategies.

Ethanol and acetaldehyde are also associated with intestinal barrier disruption through dysregulation of occludin, claudins, and zonula occludens-1 (ZO-1) at the apical junctional complex [13,29]. Mucus integrity and antimicrobial peptide function, including REG3γ and α-defensins, are compromised in parallel, weakening the separation between luminal microbes and the epithelium [13,31]. The downstream consequence is increased movement of microbial-associated molecular patterns, particularly lipopolysaccharide (LPS), toward the portal circulation [11,12,32]. In the liver, these signals engage innate immune sensors and contribute to steatohepatitis-associated inflammatory and fibrogenic signaling [8,33]. Eom et al. [33] further reported gut microbiota-associated natural killer cell activation in an experimental ALD setting, supporting the relevance of gut-derived immune readouts while remaining intervention-specific evidence.

### 3.2. Mycobiome Perturbation and Cross-Kingdom Signaling

Beyond bacterial dysbiosis, ALD has been associated with perturbation of the intestinal mycobiome, including expansion of Candida species in selected patient and experimental contexts [14,15,16]. Zeng et al. [14] reported that Candida albicans-specific Th17 responses contribute to ALD, and Chu et al. [15] showed that the C. albicans exotoxin candidalysin can promote ALD disease in experimental settings. Systemic exposure to fungal antigens or cell-wall components has also been linked to ALD severity, although these biomarkers require cautious interpretation [34,35,36].

Bacterial and fungal communities are interdependent. Bacterial organic acids, antimicrobial peptides, and other metabolic outputs can shape fungal niches, while fungal cell-wall material and metabolites may influence microbial community structure and host immune signaling [16,36]. Jiang et al. [35] framed the gut mycobiome as an emerging player in chronic liver diseases, and Zeng and Schnabl [36] summarized the broader implications of gut mycobiome alterations for liver disease. Whether the sheep yogurt matrix features, including fermentation-derived organic acids, ovine milk lipid components, and LAB-associated ecology, interact with bacterial-fungal dynamics in ALD-relevant systems remains untested.

### 3.3. Bile Acid Signaling, AhR-Tryptophan Axis, and Nutritional Vulnerability

A third cluster operates at the interface of microbial metabolism and host signaling. Alcohol exposure is associated with disruption of bile acid signaling along the farnesoid X receptor (FXR)-fibroblast growth factor 15/19 (FGF15/19) axis and through Takeda G-protein receptor 5 (TGR5) [17,18,19]. These pathways regulate hepatic bile acid synthesis, intestinal barrier maintenance, and metabolic homeostasis, although bile acid changes vary across models [37,38]. Van Best et al. [39] showed that bile acids shape gut microbiota maturation, while Gadaleta et al. [40] and Padro et al. [41] highlighted microbial bile salt hydrolase activity and selected Lactiplantibacillus plantarum strains as routes for modifying bile acid pools. These findings support bile acid profiling as a readout, not a presumed direction of effect for sheep yogurt.

ALD is also linked to altered microbial tryptophan metabolism and AhR ligand availability at the intestinal interface [20,21,42]. Wrzosek et al. [21] showed that microbiota-derived tryptophan metabolites can activate AhR signaling and improve alcohol-induced liver injury in experimental models, while Scott et al. [43] showed that microbial tryptophan metabolites regulate gut barrier function through AhR. Stockinger et al. [44] reviewed AhR as a safeguard of intestinal barrier function and IL-22-mediated antimicrobial defense, with REG3-related responses providing downstream mucosal readouts. However, AhR biology is context-dependent, with responses shaped by ligand structure, tissue context, and inflammatory state [45,46,47].

Nutritional vulnerability is superimposed on these signaling perturbations. Zinc deficiency is described in chronic alcohol use and experimental ALD and is mechanistically linked to barrier integrity, antimicrobial defense, and hepatic homeostasis [13,48]. Other deficits, including methionine-cycle and B-vitamin-related disturbances, may further compound gut-liver vulnerability [22,23,24,49]. Whether sheep yogurt-derived metabolites, microbial transformations of substrate-supplied tryptophan, matrix-delivered zinc, or proteolysis-generated peptides engage these axes remains an empirical question.

## 4. Sheep Yogurt as an Ovine-Derived Fermented Dairy Matrix

### 4.1. Food-Matrix Science and Matrix-Level Framing

In food-matrix science, a matrix is not defined simply by the list of ingredients present in a food. Rather, it refers to the physical and chemical organization of nutrients, microorganisms, metabolites, and structural components, as well as the interactions among these components during processing, digestion, and exposure to the host. A matrix effect should therefore be demonstrated by showing that an intact food produces outcomes that differ from appropriate comparators with matched or isolated components, rather than inferred from compositional differences alone.

In dairy systems, both milk species and product structure can influence digestive behavior. Comparative in vitro studies of cow and sheep milk and yogurt have reported differences in gastric disintegration, lipolysis, proteolysis, peptide release, and calcium bioaccessibility between species and between milk and yogurt structures [50,51]. These findings support the proposition that ovine substrate and fermentation structure can alter digestive exposure. However, they do not demonstrate matrix-specific physiological activity in ALD, nor do they establish that sheep yogurt improves any ALD-related outcome.

Within the present review, sheep yogurt is therefore treated as a candidate ovine-derived fermented dairy matrix rather than as an established intervention. This framing requires explicit comparator-controlled testing: sheep yogurt should be compared with unfermented sheep milk to evaluate fermentation-related differences; with matched bovine or goat yogurt to assess substrate-related differences; and with viable-cell, heat-inactivated, or component preparations to distinguish food-matrix delivery from microbial or isolated-component effects. Until such comparisons are performed in ALD-relevant systems, evidence from peptides, lipids, minerals, probiotics, postbiotics, or non-ovine fermented dairy products remains indirect or mechanistic inference.

### 4.2. Ovine Milk Substrate and Fermentation Transformations

Ovine milk differs from bovine milk in composition, although the magnitude of these differences depends on breed, lactation stage, season, feeding system, and analytical method. Compared with bovine milk, ovine milk generally has higher total solids, greater protein and fat density, and distinctive casein, whey protein, lipid, and mineral profiles [1,2]. Its lipid fraction includes a relevant medium-chain fatty acid component, and its mineral composition, including calcium, phosphorus, and zinc, varies across production and analytical contexts [1,4]. Selected compositional characteristics relevant to controlled fermented-dairy comparator design are summarized in Table 3.

Boukria et al. [1] compared yogurts prepared from different mammalian milks, providing a compositional basis for controlled fermented-dairy comparisons. Zhu et al. [2] further characterized proteomic differences among sheep, goat, and cow milk, supporting the need to avoid treating ruminant milk substrates as interchangeable. Non-ALD intestinal studies using sheep milk or sheep-milk-derived materials provide additional context for microbiota and inflammatory readouts, but they remain indirect evidence for the present ALD framework [3,52,53].

Fermentation by Streptococcus thermophilus and Lactobacillus delbrueckii subsp. bulgaricus, with or without adjunct cultures, transforms ovine milk through acidification, proteolysis, microbial metabolism, and matrix restructuring. Lactose is partially converted to lactate, while starter-culture proteolysis releases peptides from casein and whey proteins; peptide sequence and yield depend on substrate composition, strain activity, fermentation duration, storage, and digestion [6,7,54,55,56]. Pipaliya et al. [55] identified bioactive peptides from sheep milk fermented with Limosilactobacillus fermentum, and Ramos et al. [54] examined bioactive and antioxidant properties of sheep’s milk yogurt after in vitro digestion and fermentation, supporting the need to consider digestion-stage matrix outputs rather than product composition alone.

Fermentation may also alter the free fatty acid pool, matrix viscosity, and release kinetics of bioactive components. Strain-dependent exopolysaccharide production can influence matrix structure and local luminal conditions during digestion [6,7]. Nielsen et al. [56] showed that chemically acidified, live, and heat-inactivated fermented dairy yogurts differ in peptide, amino acid, and small-compound profiles, reinforcing the need to treat fermentation state and viable-cell status as experimentally distinct variables. The resulting product is therefore not “ovine milk plus a probiotic,” but a substrate-specific, strain-specific, and structure-specific matrix.

### 4.3. Distinction from Generic Fermented Dairy and Other Comparators

Treating sheep yogurt as a distinct object of inquiry requires explicit non-equivalence with common proxies. Generic fermented dairy is a broad category rather than a defined product class. Bovine yogurt differs from sheep yogurt in protein density, casein and whey composition, fatty acid profile, mineral distribution, and matrix behavior; these differences may propagate into acidification kinetics, peptide profiles, digestion behavior, and component release [1,2,5]. Bovine yogurt evidence, therefore, remains indirect for ovine-derived fermented matrices.

Goat yogurt and goat milk products are closer comparators but still non-equivalent. Goat and sheep dairy matrices overlap within the small-ruminant frame, yet differ in protein composition, lipid structure, digestibility, and matrix properties [1,4,57,58]. Chen et al. [5] reviewed fermented goat dairy products in comparison with fermented cow milk, supporting goat yogurt as a comparator rather than a substitute for sheep yogurt. Goat-dairy studies involving intestinal barrier, microbiota, SCFA-related, exosome, or aging readouts provide useful indirect context, but they remain caprine, non-ALD, and product-specific evidence [52,59,60,61,62].

Other comparators isolate different matrix axes. Unfermented ovine milk lacks the fermentation-related changes in acidification, proteolysis, viable LAB delivery, microbial metabolism, and postbiotic-like outputs [56,63]. Probiotic capsules separate viable microorganisms from substrate and fermentation metabolites, whereas heat-inactivated or postbiotic-like preparations retain selected microbial or fermentation-derived material but lack viable-cell activity [56,64,65,66]. Treven et al. [64] showed that the food matrix can affect probiotic survival during simulated digestion, supporting the relevance of matrix delivery in future comparator design. Postbiotic studies in epithelial or ALD-related systems may guide comparator arms, but they do not define the activity of intact sheep yogurt [63,65].

Fermented dairy evidence is also product-dependent. Studies on kefir, acid whey, fermented dairy lactobacilli, and functional yogurt support the broader plausibility for fermented food research, but they cannot be transferred directly to sheep yogurt in ALD-relevant systems [7,67,68,69]. Each comparator therefore isolates one axis-substrate, fermentation, viable cells, postbiotic-like material, isolated components, or matrix structure and none is interchangeable with sheep yogurt itself. The relevant question is not whether sheep yogurt is inherently superior to other fermented dairy products, but whether its matrix properties produce measurable differences in ALD-relevant gut-liver axis readouts under controlled comparator conditions.

## 5. Mechanistic Hypotheses Linking Sheep Yogurt Matrix Features to ALD Gut-Liver Axis Nodes

**Figure 2:**
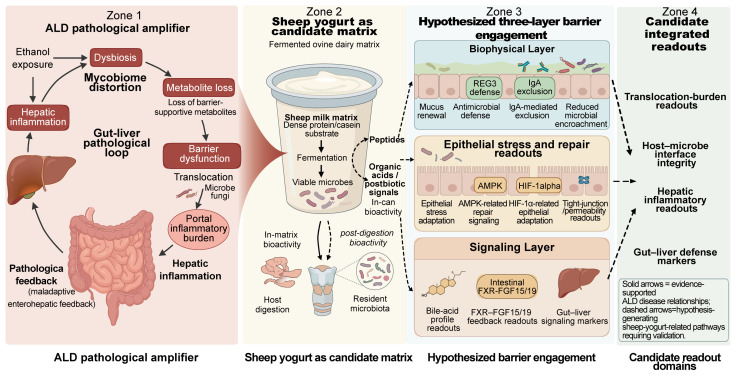
Evidence-bounded conceptual framework linking ALD gut-liver axis injury to hypothesized sheep yogurt matrix engagement points. This conceptual, non-quantitative schematic summarizes ALD-associated gut-liver axis amplification and maps sheep yogurt as a candidate ovine-derived fermented dairy matrix to hypothesized biophysical, biochemical, and signaling engagement layers. Solid arrows indicate evidence-supported relationships involved in ALD gut-liver axis injury. Dashed arrows and the dashed boundary surrounding Zone 3 denote hypothesized sheep yogurt-related engagement pathways and candidate readout mappings that require experimental validation. The colored bands in Zone 3 distinguish biophysical, epithelial stress-and-repair, and signaling domains only; they do not indicate evidence strength or direction of effect. Boxes in Zone 4 represent candidate outcome measures for testing these hypotheses.

ALD mechanisms are summarized only as a disease-context framework for selecting experimental readouts. It does not establish that sheep yogurt engages these pathways. The following biophysical, biochemical, and signaling layers are organized as testable matrix hypotheses: for each layer, the relevant question is whether an intact sheep yogurt preparation differs from defined comparators in ALD-relevant systems. Figure 2 presents the evidence-bounded conceptual framework linking ALD-related gut-liver axis injury with candidate sheep yogurt matrix inputs and corresponding testable readout domains.

### 5.1. Biophysical Layer: Mucus, Barrier, and Microbial Exclusion

ALD is associated with thinning of the colonic mucus layer, dysregulation of occludin, claudins, and zonula occludens-1 (ZO-1), attenuation of antimicrobial peptide defenses including REG3γ and α-defensins, and failure of microbial exclusion at the epithelial interface [13,31,32]. These abnormalities create a biophysical vulnerability in which the separation between luminal microbes and the host epithelium becomes compromised.

Dairy-matrix studies provide a basis for testing, but not presuming, barrier-related effects. The protein-lipid network, gel structure, and digestion kinetics of yogurt can influence the release and availability of matrix components during gastrointestinal transit [50,51,63,64]. Likewise, viable LAB, exopolysaccharides, organic acids, and proteolysis-derived peptides represent experimentally separable features of a fermented dairy product [7,69,70,71,72]. No study has shown that these features, when delivered as sheep yogurt, improve mucus integrity, epithelial barrier function, or microbial exclusion in ALD. They should therefore be used to define comparator arms and barrier-related readouts, rather than to predict a beneficial effect.

**Figure 3:**
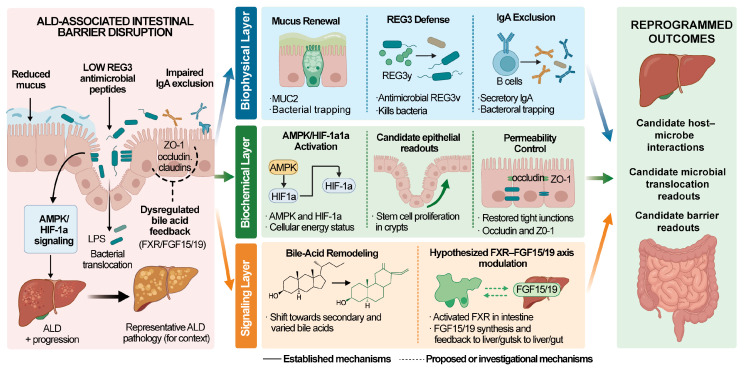
Candidate barrier-related readout domains for ALD gut-liver axis studies. This conceptual, non-quantitative figure summarizes key barrier-related readout domains in ALD gut-liver axis research, including mucus renewal, antimicrobial defense, IgA exclusion, epithelial repair, permeability control, bile acid remodeling, and FXR–FGF15/19 feedback. Solid black arrows and lines indicate established ALD-related mechanisms or pathological relationships, whereas dashed lines indicate proposed or investigational mechanistic links requiring validation. Blue, green, and orange arrows map the biophysical, biochemical, and signaling readout layers, respectively, to candidate outcome domains; these colors distinguish conceptual layers only and do not indicate evidence strength or direction of effect. The reprogrammed outcomes shown on the right are candidate host–microbe, microbial-translocation, and barrier-related readouts for future studies of fermented dairy matrices. They do not represent demonstrated effects of sheep yogurt. The figure serves as a mechanistic readout map rather than evidence of therapeutic activity.

These indirect studies support the selection of mucus, tight-junction, permeability, antimicrobial peptide, secretory IgA, and translocation markers as assay targets. Candidate barrier-related readout domains are summarized in Figure 3. They do not, however, establish that sheep yogurt improves barrier function in ALD-relevant systems.

### 5.2. Biochemical Layer: Epithelial Stress, Peptides, MCFA, and Zinc

A second layer concerns the biochemical environment in which the epithelium operates under chronic ethanol exposure. Acetaldehyde- and ethanol-related oxidative stress can destabilize tight-junction architecture, impair epithelial repair, and alter AMPK- and HIF-1α-related homeostatic signaling [13,73,74,75]. Zinc-related vulnerability adds another biochemical dimension because zinc-dependent processes contribute to epithelial integrity, antimicrobial defense, and hepatic homeostasis [13,48].

Fermentation and gastrointestinal digestion can generate peptide profiles that differ according to milk species, starter culture, and product structure [50,51,54,55,56]. Studies of fermented sheep-milk products have characterized peptides and in vitro antioxidant-related properties, but these findings do not establish epithelial repair, oxidative-stress reduction, or liver protection in ALD [54,55]. Their value in the present framework is limited to identifying analytes and comparator conditions for future testing, including peptide profiling of intact sheep yogurt and unfermented sheep milk, as well as matched fermented dairy comparators.

The lipid and mineral fractions should also be evaluated cautiously. MCFA supplied by ovine milk lipids may be relevant to microbial niche conditions, but matrix-delivered MCFA cannot be equated with isolated lipid exposure without direct comparison [1,4]. Zinc and other minerals are co-digested with peptides and lipids; therefore, the biological relevance of matrix-delivered zinc should be evaluated separately from isolated zinc supplementation evidence [6,13,48,56]. Appropriate readouts include TEER, FITC-dextran flux, tight-junction protein localization, oxidative stress markers, AMPK/HIF-1α-related signaling, zinc status, metallothionein expression, and epithelial repair kinetics.

### 5.3. Signaling Layer: Bile Acid Signaling, AhR-Tryptophan Axis, and Microbial Metabolites

A third layer concerns signaling networks through which microbial metabolites and dietary substrates communicate with epithelial and hepatic compartments. Alcohol exposure is associated with disruption of bile acid signaling along the FXR-FGF15/19 axis and through TGR5 [17,18,19]. These pathways regulate hepatic bile acid synthesis, intestinal barrier maintenance, and metabolic homeostasis, although the direction and magnitude of bile acid changes vary across models [37,38]. Microbial bile salt hydrolase activity and selected Lactiplantibacillus plantarum strains have been discussed as routes for modifying bile acid pools, supporting bile-acid profiling as a readout rather than a presumed direction of benefit [40,41].

The AhR-tryptophan axis provides a second signaling domain. ALD is linked to altered microbial tryptophan metabolism and AhR ligand availability at the intestinal interface [20,21,42]. Wrzosek et al. [21] showed that microbiota-derived tryptophan metabolites can activate AhR signaling and improve alcohol-induced liver injury in experimental models, while Scott et al. [43] showed that microbial tryptophan metabolites can regulate gut barrier function through AhR. Stockinger et al. [44] reviewed AhR as a safeguard of intestinal barrier function, and IL-22-mediated antimicrobial defense with REG3-related responses provides downstream mucosal readouts [31].

The presence of viable LAB, fermentation-derived metabolites, substrate-supplied tryptophan, and minerals in sheep yogurt provides a rationale for measuring bile-acid and tryptophan-related pathways, but does not establish that the product modifies these pathways in vivo. Although some LAB strains have bile salt hydrolase activity and microbial tryptophan metabolites can influence AhR signaling in other contexts [40,41,42,43,76,77], no evidence currently shows that sheep yogurt changes bile-acid pools, indole availability, AhR activity, or downstream mucosal responses in ALD. These variables should therefore be considered exploratory mechanistic endpoints in comparator-controlled studies.

Probiotic and postbiotic studies in ALD-related systems reinforce the importance of strain, product format, and comparator design. Representative studies have examined multi-strain probiotics, postbiotic products, fermented milk, compound probiotics, and strain-specific effects on barrier integrity, microbiota, bile acids, AMPK signaling, inflammatory signaling, or ferroptosis-related mechanisms [78,79,80,81,82]. These findings support microbial-intervention comparator logic, but they should not be treated as direct evidence for sheep yogurt.

### 5.4. Integrated Matrix-Level Model

The three layers provide an organizing framework for experimental design rather than a model of demonstrated sheep yogurt action. Sheep yogurt contains a physical dairy structure, viable LAB, fermentation-derived metabolites, peptides, lipids, minerals, and postbiotic-like material; however, the contribution of each feature, alone or in combination, to ALD-relevant biology is unknown. The matrix-level proposition is limited to the following testable question: does an intact, well-characterized sheep yogurt preparation differ from appropriate dairy, microbial, and component comparators in predefined ALD-relevant readouts? The present evidence does not support the expectation that sheep yogurt restores barrier function, reduces microbial or fungal translocation, modifies bile-acid or AhR signaling, or improves ALD outcomes. Table 4 summarizes the candidate matrix inputs, ALD-relevant outcome measures, and representative supporting evidence that can guide such comparator-controlled studies.

## 6. Research Framework for Comparator-Controlled Evaluation

**Figure 4:**
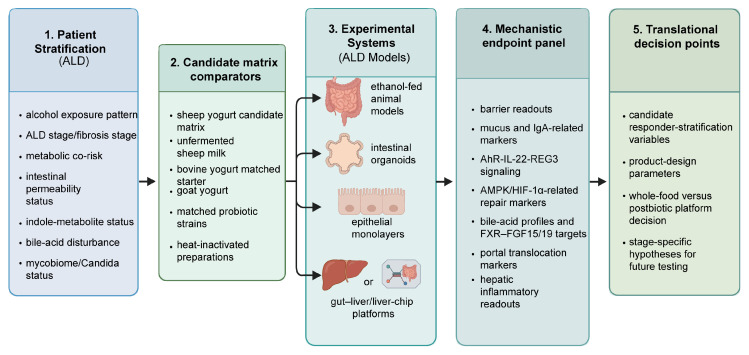
Translational research framework for comparator-controlled evaluation of sheep yogurt in ALD-related gut-liver axis models.

The hypotheses developed above require designs that can distinguish sheep yogurt-specific signals from generic fermented-dairy effects, probiotic effects, postbiotic-like effects, and isolated-component effects. Figure 4 summarizes a translational framework organized around model- or patient-stratification, comparator selection, ALD-relevant experimental systems, mechanistic endpoints, and decision criteria. The purpose is not to infer efficacy, but to specify how the matrix-level hypotheses in Chapter 5 could be tested.

ALD-related gut-liver axis injury is heterogeneous across disease stage, microbial ecology, barrier status, and nutritional state [10,11,29]. Malnutrition severity, infectious outcomes, sex, metabolic co-risk, and alcohol exposure pattern may further influence translational interpretation and study design [26,83,84,85,86]. These variables should inform model selection, biomarker interpretation, and stratified analysis rather than being treated as secondary descriptors.

Experimental systems should be selected according to the mechanism layer under study. Ethanol-fed animal models remain necessary for integrated in vivo readouts, whereas epithelial monolayers, intestinal organoids, liver organoids, gut-liver co-cultures, and Liver-Chip systems can help isolate barrier, metabolic, and hepatic interface mechanisms before animal or clinical testing [87,88,89,90,91]. Nawroth et al. [87] modeled ALD in a human Liver-Chip, and Ariño et al. [88] used patient-derived liver organoids to model ALD. Broader organoid platforms also support disease modeling and translational screening, but they should not be treated as substitutes for ALD-specific in vivo validation [91,92,93,94,95,96].

### 6.1. Evidence Boundary at the Start of the Agenda

Direct evidence for sheep yogurt in ALD-relevant experimental or clinical systems remains absent, and the agenda below cannot support dietary recommendations or clinical claims. Its function is to define the conditions under which matrix-level attribution would become possible.

This boundary also prevents overinterpretation of adjacent evidence. A positive result from a probiotic capsule would not establish a fermented-food-matrix effect; a positive result from an isolated peptide, MCFA, or zinc preparation would not establish an intact-yogurt effect; and a compositional distinction among sheep, bovine, and goat milk would not by itself establish disease relevance. Matrix attribution requires comparator-controlled testing in ALD-relevant systems.

### 6.2. Experimental Systems Matched to Mechanism Layer

System choice should follow the hypothesis under test. For the biophysical layer, alcohol-exposed epithelial monolayers, mucus-producing co-cultures, intestinal organoids, and alcohol-fed animal models can support readouts of mucus integrity, tight junction architecture, permeability, microbial exclusion, secretory IgA, portal translocation markers, and hepatic inflammatory response [13,32,87,96]. For the biochemical layer, acetaldehyde- or ethanol-challenged epithelial systems, oxidative stress models, and zinc-deficient or zinc-replete designs can be used to examine TEER, FITC-dextran flux, oxidative stress markers, AMPK/HIF-1α-related signaling, zinc status, metallothionein expression, and epithelial repair [13,48,73,74].

Signaling-layer hypotheses require models that allow simultaneous measurement of bile acid pools, microbial metabolite profiles, and host signaling readouts. Alcohol-fed rodent models with paired stool, portal blood, intestinal tissue, and hepatic tissue can support analysis of bile acid profiles, FXR and TGR5 target genes, FGF15 or FGF19, indole metabolites, AhR target genes, IL-22, and REG3γ [17,18,19,20,44]. Gut-liver co-cultures and Liver-Chip platforms may complement in vivo models when directional contributions of specific matrix inputs need to be isolated [87,90,92]. Any signal observed in simplified systems should be interpreted against comparator arms that distinguish intact sheep yogurt from unfermented sheep milk, isolated components, or heat-inactivated preparations.

### 6.3. Comparator-Controlled Designs for Matrix Attribution

Comparator-controlled designs should be organized by the matrix axis under test rather than by intervention labels alone. Sheep yogurt versus unfermented sheep milk isolates the fermentation contribution on a shared ovine substrate [55,56,63]. Sheep yogurt versus bovine yogurt, prepared with matched starter cultures, isolates the ovine substrate contribution relative to generic fermented dairy [1,2,5]. Sheep yogurt versus goat yogurt distinguishes ovine-specific features from broader small-ruminant dairy properties [1,4,57]. Same-strain probiotic and heat-inactivated comparators help separate food-matrix delivery, viable-cell activity, and postbiotic-like contributions [56,64,65,66,69]. Comparisons with isolated MCFA, peptide fractions, zinc, or component cocktails can test whether the intact matrix differs from the sum of selected components [4,40,48,55].

No single contrast can establish clinical benefit. The purpose of these comparisons is matrix attribution: to determine whether an observed signal is linked to fermentation, ovine substrate, viable-cell delivery, postbiotic-like material, isolated components, or the intact matrix. Studies should pre-specify the matrix axis under test, the readouts that define success or failure, and whether the outcome is barrier-related, microbial, fungal, signaling-related, nutritional, or hepatic. Representative evidence streams supporting these comparator decisions are summarized in Supplementary Table S2.

### 6.4. Biomarker Panels, Product Standardization, and Stratification

Biomarker reporting should map onto the mechanism layers developed in Chapter 5 rather than rely on a single favorable endpoint. Barrier and translocation readouts should include mucus, tight-junction, permeability, microbial, fungal, and portal exposure markers, with careful selection of permeability endpoints supported by established measurement frameworks [14,16,32,34,97]. Bacteriome and mycobiome readouts should include sequencing-based profiling and functional outputs such as SCFA and lactate, while microbial biomarkers may support stratification and response tracking in ALD-oriented designs [98]. Signaling, nutritional, and hepatic-interface readouts should include bile acid profiles, FXR/TGR5 targets, FGF15/19, indole metabolites, AhR-related markers, IL-22, REG3γ, zinc status, oxidative stress markers, ALT, AST, hepatic cytokines, and histological outcomes.

Product standardization is equally important. Sheep yogurt preparations should report sheep breed, lactation stage, season, feeding system, milk composition, starter strain identity, adjunct cultures, fermentation conditions, pH, titratable acidity, viable counts, peptide profile where possible, MCFA profile, zinc and mineral content, exopolysaccharide production, storage conditions, and digestion or exposure preparation. Without these data, the results cannot be attributed to an ovine-derived fermented dairy matrix in a reproducible way.

Stratification should be built into both preclinical and translational designs. Relevant axes include alcohol exposure pattern, ALD stage, intestinal permeability, microbiome and mycobiome structure, bile acid disturbance, indole metabolite status, zinc status, protein-energy malnutrition, sex, metabolic co-risk, and medication exposure [83,84,85,86,99]. These variables may determine whether a fermented matrix survives transit, releases components, interacts with the damaged gut environment, or produces measurable readouts.

### 6.5. Safety Considerations and One Health-Oriented Translational Pathway

Translational planning cannot be separated from safety considerations. Live-microorganism exposure carries different safety considerations in advanced liver disease, where bacterial translocation, immune dysfunction, and infection risk may alter the risk profile of viable LAB ingestion [11,100]. Where live-cell exposure is not appropriate, heat-inactivated or postbiotic-like preparations may serve as comparator arms, but they should not be treated as substitutes for sheep yogurt; their purpose is to isolate viable-cell contribution from inactivated microbial material and accumulated fermentation products.

The framework outlined here is consistent with a One Health perspective in which ovine dairy production, fermented food-matrix standardization, animal nutrition, and gut-liver axis research are addressed within a single agenda. The ovine substrate is shaped by upstream veterinary dairy practices, the matrix is constructed by fermentation microbiology, and the candidate readouts sit in gut-liver axis biology. This integration supports structured evaluation rather than clinical recommendation.

## 7. Discussion: Gaps, Controversies, and Methodological Needs

The preceding chapters developed a matrix-level framework in which sheep yogurt is treated as an ovine-derived fermented dairy matrix and ALD as a representative gut-liver axis disorder. This discussion highlights three issues that must be resolved before the framework can be advanced experimentally: direct evidence gaps, unresolved controversies in adjacent literature, and methodological requirements for matrix-level attribution.

### 7.1. Research Gaps

The decisive gap is the absence of direct evidence. Studies testing sheep yogurt or a defined sheep yogurt preparation in ALD-relevant experimental or clinical systems are currently lacking, and matched comparisons among sheep yogurt, bovine yogurt, goat yogurt, and unfermented sheep milk remain essentially undocumented. As a result, the substrate-versus-fermentation contributions distinguished in this review remain operational hypotheses.

A second gap concerns exposure. The dose-exposure-effect chain connecting realistic intake of an ovine-derived fermented matrix to luminal, portal, and hepatic readouts has not been characterized. Evidence for matrix-delivered MCFA, casein- and whey-derived peptides, fermentation metabolites, and zinc remains dominated by bovine-substrate or non-ALD systems [1,54,55,56]. Sheep milk and sheep yogurt in non-ALD intestinal inflammation models provide useful context, but they cannot substitute for testing sheep yogurt in ALD-relevant systems [3,52,53,54].

A third gap lies upstream, at the dairy-science end of the chain. Breed, feeding system, lactation stage, season, starter culture identity, fermentation endpoint, and storage conditions are rarely connected to gut-liver axis outcomes. Without these variables, future biological signals cannot be confidently attributed to the ovine substrate, fermentation process, viable microbial delivery, postbiotic-like factors, or intact matrix.

Adjacent fermented milk studies in colitis and intestinal inflammation illustrate the value and limits of indirect evidence: they support assay selection and product-design thinking, but remain non-ALD and non-sheep yogurt evidence [101,102,103,104,105].

### 7.2. Scientific Controversies

Several assumptions often used to support fermented dairy interventions require caution. First, yogurt-derived lactate should not be treated as equivalent to fiber-derived SCFAs, because lactate kinetics, microbial cross-feeding, and downstream conversion differ from acetate, propionate, and butyrate [106,107]. Second, the presence of C8:0 and C10:0 in ovine milk supports compositional comparison, but does not establish in vivo fungal niche restriction from a fermented dairy matrix at realistic luminal exposures [1,4]. Third, bile acid signaling is not directionally simple: FXR, TGR5, and FGF15/19-related changes vary across ALD models and should be interpreted through downstream receptor, barrier, microbial, and hepatic readouts rather than assumed to be beneficial [17,18,19,37,108].

The AhR pathway requires similar caution. AhR biology depends on ligand structure, dose, exposure duration, cell type, inflammatory context, and downstream target selection [44,45,46,47]. No study has demonstrated that sheep yogurt modifies indole production, AhR activity, IL-22, REG3γ, or related mucosal responses in ALD-relevant systems. Accordingly, these variables should be regarded as hypothesis-testing readouts rather than anticipated biological responses. Likewise, probiotic, postbiotics, fermented milk, and intact fermented food-matrix evidence are not interchangeable; each category differs in live-cell delivery, inactivated microbial material, fermentation metabolites, food-carrier effects, and whole-matrix interactions [6,56,64].

### 7.3. Limitations of Extrapolating Adjacent Evidence to Human ALD

A major limitation of the current evidence base is the need to extrapolate from animal models and non-ALD systems. Ethanol exposure regimen, diet composition, disease stage, host genetics, intestinal anatomy, microbial ecology, immune responses, and hepatic metabolism differ substantially between experimental models and human ALD. Consequently, a favorable or unfavorable result in a rodent ethanol model cannot establish the direction, magnitude, or clinical relevance of an effect in patients with ALD.

Evidence from colitis, intestinal inflammation, simulated digestion, epithelial-cell systems, and non-ALD metabolic models is similarly informative only at the level of biological plausibility or assay selection [3,52,53,54,101,102,103,104,105]. These models may help identify candidate endpoints, such as permeability, microbial metabolites, or inflammatory markers, but they do not demonstrate that sheep yogurt improves ALD-related outcomes in humans. Differences in disease trigger, alcohol exposure, nutritional status, medication use, and baseline microbiome composition further limit direct transferability. Therefore, all adjacent evidence synthesized in this review should be interpreted as indirect or mechanistic inference, rather than as evidence of clinical benefit.

### 7.4. Product and Translational Limitations of Sheep Yogurt as a Nutritional Intervention

Sheep yogurt is not a uniform intervention. Its composition may vary according to breed, feeding system, lactation stage, milk processing, starter-culture identity, fermentation conditions, fermentation endpoint, storage duration, and manufacturing practices [1,2,54,55,56]. These variables can affect nutrient composition, peptide and metabolite profiles, viable microbial counts, acidity, texture, and digestion behavior. Without rigorous product characterization and batch-level standardization, any observed biological signal cannot be confidently attributed to sheep yogurt as a defined matrix.

Translational applicability also remains uncertain. The feasibility, tolerability, dose, frequency of intake, dietary context, and safety profile of sheep yogurt require evaluation in clinically relevant ALD populations, particularly across differing disease stages and nutritional states. Its status as a food product does not, by itself, establish suitability as an adjunctive nutritional intervention. Future clinical studies should therefore assess product acceptability, adherence, safety, and clinically meaningful outcomes alongside mechanistic endpoints.

### 7.5. Methodological Needs

Five methodological requirements follow from these gaps and controversies. First, substrate-side and product-side standardization must be treated as core experimental metadata, including breed, feeding system, lactation stage, milk composition, starter identity, fermentation conditions, viable counts, peptide profile, MCFA profile, mineral content, and storage. Second, digestion state should be specified, because whole yogurt, simulated gastrointestinal digests, sterile filtrates, lipid fractions, peptide fractions, viable-cell preparations, and heat-inactivated preparations may produce different exposure profiles; standardized digestion protocols are therefore needed [63,64].

Third, matrix attribution requires comparator-controlled design. The central question is not whether sheep yogurt differs from an untreated control, but whether a signal can be assigned to fermentation, ovine substrate, small-ruminant matrix features, food-matrix delivery, viable-cell activity, postbiotic-like material, or intact-matrix interactions. Fourth, biomarker panels should be pre-specified and anchored to barrier, translocation, bile acid, AhR-tryptophan, nutritional, and hepatic endpoints rather than interpreted retrospectively. Fifth, stratification and safety should be built into the design from the beginning, because disease stage, alcohol exposure, microbiome and mycobiome structure, nutritional status, sex, metabolic co-risk, medication exposure, and live-microorganism risk may all affect interpretation [11,26,100].

### 7.6. Future Directions and One Health Relevance

Future work should retain the matrix-level framing rather than re-aggregating sheep yogurt with generic fermented dairy. Ovine dairy production, animal nutrition, fermentation microbiology, product standardization, and gut-liver axis biology should be addressed as a connected research agenda. Upstream determinants of the matrix—including breed, feeding system, lactation stage, milk composition, starter culture identity, and fermentation endpoint—should be reported as potential sources of variability in health-related outcomes.

The One Health relevance of this agenda lies in connecting veterinary dairy science with host-microbe metabolic health research. Sheep yogurt is shaped by animal production systems, transformed by fermentation microbiology, and evaluated through host gut-liver axis readouts. This makes it a useful model for cross-disciplinary research, but not a clinical recommendation. The appropriate scientific posture remains structured evaluation rather than extrapolation.

## 8. Conclusions

This scoping review provides no evidence for sheep yogurt efficacy in ALD. It proposes an evidence-bounded framework that treats sheep yogurt as a distinct ovine matrix, distinct-discrete from other fermented dairy, probiotic, and isolated components, and prioritizes gut-liver axis pathways for hypothesis testing. Direct evidence remains absent. Thus, no clinical or dietary recommendation can be made. Future studies demand standardized preparations, comparator-controlled designs, and predefined outcomes; sheep yogurt, pending validation, is a conceptual research model rather than an established intervention.

## Figures and Tables

**Table 1:** Evidence domains and search concepts used in the PRISMA-ScR-guided scoping review.

Evidence Domain	Core Search Concepts	Role in the Review
ALD and ethanol-related liver injury	Alcohol-associated liver disease, alcohol-related liver disease, alcoholic liver disease, alcoholic hepatitis, ethanol-induced liver injury	Defined the disease context and identified ALD-relevant experimental and clinical evidence.
Gut-liver axis dysfunction	Gut–liver axis, intestinal barrier, permeability, tight junctions, mucus, dysbiosis, microbiota, mycobiome, microbial/fungal translocation, LPS, endotoxemia	Identified evidence on barrier injury, microbial ecology, and gut-derived inflammatory exposure.
Metabolic, immune, and nutritional pathways	Bile acids, FXR, TGR5, FGF15/19, tryptophan, indoles, AhR, zinc, malnutrition, sarcopenia	Identified pathway-based evidence and candidate mechanistic endpoints.
Fermented dairy and microbial interventions	Yogurt, fermented milk, kefir, probiotics, postbiotics, lactic acid bacteria	Identified adjacent evidence from fermented dairy, probiotic, and postbiotic interventions.
Ovine dairy and sheep yogurt matrix	Sheep milk, ewe milk, ovine milk, sheep/ovine yogurt, fermentation, digestion, food matrix	Identified evidence on ovine substrate composition, fermentation-related features, and matrix characteristics.
Components and comparator evidence	Peptides, medium-chain fatty acids, zinc, minerals, viable cells, heat-inactivated preparations, bovine and goat dairy	Supported comparator selection and distinction between intact sheep yogurt and isolated components or related products.
Targeted direct-evidence search	Sheep/ovine yogurt or milk combined with ALD, ethanol injury, gut-liver axis, barrier, microbiome, bile acid, or AhR terms	Determined whether sheep yogurt had been directly tested in ALD-relevant systems.
Manual searching and citation tracking	Reference lists, backward and forward citation tracking	Identified additional relevant mechanistic, comparator, and matrix-related sources.

**Table 2:** Evidence-classification framework and interpretation boundaries.

Evidence Category	What Was Included	Role in the Review	Interpretation Boundary
Direct evidence	Studies testing sheep yogurt or a defined sheep yogurt preparation in ALD, ethanol-induced liver injury, alcoholic hepatitis, or ALD-relevant gut–liver axis systems	Determined whether direct evidence for sheep yogurt in ALD exists	Absence of direct evidence represents an evidence gap, not evidence of no effect.
Indirect evidence: ALD and gut–liver context	Clinical, animal, and mechanistic studies of ALD, barrier dysfunction, dysbiosis, mycobiome alterations, microbial translocation, malnutrition, and inflammation	Defined disease relevance and priority outcome domains	Disease relevance does not demonstrate sheep yogurt efficacy.
Indirect evidence: fermented dairy and microbial interventions	Studies of yogurt, fermented milk, kefir, probiotics, postbiotics, lactic acid bacteria, or microbial preparations	Informed comparator logic and candidate intervention mechanisms	Product-specific findings cannot be generalized to intact sheep yogurt.
Indirect evidence: ovine dairy matrix	Studies of sheep milk, ovine yogurt, fermentation, digestion, peptides, minerals, and food-matrix characteristics	Supported treatment of sheep yogurt as a distinct ovine-derived matrix	Compositional or in vitro evidence does not establish ALD-related benefit.
Mechanistic inference	Epithelial, organoid, gut–liver, metabolic, nutritional, or pathway studies relevant to barrier function, bile acids, AhR signaling, oxidative stress, or immunity	Generated hypotheses and identified measurable endpoints	Pathway plausibility does not establish disease-modifying or clinical efficacy.
Comparator evidence	Studies comparing ovine, bovine, caprine, fermented, unfermented, probiotic, postbiotic, or isolated-component interventions	Guided future comparator-controlled experimental designs	Comparative evidence supports attribution logic, not sheep yogurt efficacy claims.

**Table 3:** Selected compositional characteristics of sheep, cow, and goat milk relevant to fermented-dairy comparator design.

Matrix Feature	Sheep/Ewe Milk	Cow/Bovine Milk	Goat Milk	References
Total protein (g/100 g)	6.21–6.30	3.23–3.50	2.90–3.83	[1]
Casein (g/100 g)	3.78–5.20	2.28–3.27	2.14–3.18	[1,2]
Calcium (mg/100 g)	195–200	112–120	126–198	[1]
Phosphorus (mg/100 g)	124–158	59–92	97–153	[1]
Zinc (mg/100 g)	5.20–7.47	0.30–3.80	0.43–3.40	[1]

Values are reported as presented in the cited studies and may vary with breed, lactation stage, season, feeding system, and analytical method. These compositional differences justify controlled comparator designs but do not establish biological superiority or ALD-related benefit.

**Table 4:** Candidate sheep yogurt matrix inputs, ALD-relevant outcome measures, and representative supporting evidence for future comparator-controlled studies.

Candidate Matrix Input	ALD-Relevant Domain	Priority Outcome Measures	Evidence Status	Representative Supporting References
Physical dairy matrix, exopolysaccharides, and viable LAB	Mucus integrity, microbial exclusion, and mucosal immune defense	MUC2, mucus thickness, goblet cells, REG3γ, secretory IgA, microbial encroachment	Hypothesis-generating; not directly tested for sheep yogurt in ALD.	[6,7,29,52,68]
Fermentation-derived peptides and matrix-delivered zinc	Tight-junction injury, epithelial stress, and repair capacity	TEER, FITC-dextran flux, occludin, claudins, ZO-1, oxidative-stress markers, AMPK/HIF-1α-related signaling	Indirect evidence from non-ALD or non-sheep yogurt systems.	[13,50,55,56,57,69,70]
Viable LAB, organic acids, and medium-chain fatty acids	Bacterial/fungal ecology and microbial translocation	Bacterial and fungal sequencing, Candida burden, β-glucan, LPS, sCD14, and LBP	Mechanistic inference; matrix-delivered effects remain unproven.	[1,4,6,7,27]
LAB metabolic activity, tryptophan substrate, and bile-acid interactions	Bile-acid signaling and the AhR–tryptophan axis	Bile-acid profiles, FXR/TGR5 targets, FGF15/19, indole metabolites, CYP1A1, IL-22, and REG3γ	Indirect pathway evidence; not established for sheep yogurt.	[20,21,42,43,44,45,74,75]
Whole-matrix exposure	Portal inflammatory burden and hepatic interface	LPS, sCD14, LBP, β-glucan, ALT, AST, hepatic cytokines, and histology	Downstream candidate outcomes only; no direct sheep yogurt ALD evidence.	[11,12,13,34,35,36]

This table defines candidate inputs, outcome measures, and representative supporting evidence for future comparator-controlled studies. The cited literature supports the selection of inputs or readouts but does not demonstrate that sheep yogurt improves any of the listed endpoints in ALD.

## Data Availability

The data presented in this study are available on request from the corresponding authors.

## Supplementary Materials

The following supporting information can be downloaded at: https://www.mdpi.com/article/10.3390/nu18152549/s1, Table S1: Complete database-specific search strategies for PubMed/MEDLINE, Web of Science, Scopus, and Google Scholar; Table S2: Representative evidence streams informing the sheep-yogurt matrix research framework.

## Abbreviations

The following abbreviations are used in this manuscript:

AhR	Aryl hydrocarbon receptor
ALD	Alcohol-associated liver disease
ALT	Alanine aminotransferase
AMPK	Adenosine monophosphate-activated protein kinase
AST	Aspartate aminotransferase
FGF15/19	Fibroblast growth factor 15/19
FITC-dextran	Fluorescein isothiocyanate-dextran
FXR	Farnesoid X receptor
HIF-1α	Hypoxia-inducible factor 1-alpha
IL-22	Interleukin-22
LAB	Lactic acid bacteria
LPS	Lipopolysaccharide
MCFA	Medium-chain fatty acids
REG3γ	Regenerating islet-derived protein 3 gamma
SCFA	Short-chain fatty acids
TEER	Transepithelial electrical resistance
TGR5	Takeda G-protein receptor 5
ZO-1	Zonula occludens-1
