# Supplementary material for: Gut–Liver Axis Dysfunction in Alcohol-Associated Liver Disease and the Potential Role of Sheep Yogurt: A Scoping Review and Mechanistic Framework
Source: Nutrients. 2026 Aug 4;18(15):2549. doi: 10.3390/nu18152549 (PMC13467697; doi:10.3390/nu18152549)
Supplement: Supplementary file 1 [file nutrients-18-02549-s001.zip › nutrients-4439773-supplementary.pdf]

Review

Supplementary Material

Supplementary Table S1. Complete database-specific search strategies for PubMed/MEDLINE, Web of Science, Scopus, and Google Scholar

All searches covered publications from January 2006 to February 2026. Google Scholar was used as a supplementary source for targeted searching and citation tracking.

Database	Search date and date range	Complete search strategy
PubMed/MED-LINE	12 February 2026; 1 January 2006–12 February 2026	((("Alcoholic Liver Diseases"[Mesh] OR "Alcohol-Related Disorders"[Mesh] OR "alcohol-associated liver disease"[Title/Abstract] OR "alcohol-related liver disease"[Title/Abstract] OR "alcoholic liver disease"[Title/Abstract] OR "alcoholic hepatitis"[Title/Abstract] OR "ethanol-induced liver injury"[Title/Abstract] OR "alcohol-induced liver injury"[Title/Abstract]) AND ("gut-liver axis"[Title/Abstract] OR "intestinal barrier"[Title/Abstract] OR "intestinal permeability"[Title/Abstract] OR "tight junction*"[Title/Abstract] OR mucus[Title/Abstract] OR dysbiosis[Title/Abstract] OR microbiota[Title/Abstract] OR microbiome[Title/Abstract] OR mycobiome[Title/Abstract] OR Candida[Title/Abstract] OR "microbial translocation"[Title/Abstract] OR "fungal translocation"[Title/Abstract] OR lipopolysaccharide[Title/Abstract] OR endotoxemia[Title/Abstract] OR "bile acid*"[Title/Abstract] OR FXR[Title/Abstract] OR TGR5[Title/Abstract] OR FGF15[Title/Abstract] OR FGF19[Title/Abstract] OR tryptophan[Title/Abstract] OR indole*[Title/Abstract] OR "aryl hydrocarbon receptor"[Title/Abstract] OR AhR[Title/Abstract] OR zinc[Title/Abstract] OR malnutrition[Title/Abstract] OR sarcopenia[Title/Abstract] OR yogurt[Title/Abstract] OR yoghurt[Title/Abstract] OR "fermented dairy"[Title/Abstract] OR "fermented milk"[Title/Abstract] OR kefir[Title/Abstract] OR probiotic*[Title/Abstract] OR postbiotic*[Title/Abstract] OR "lactic acid bacteria"[Title/Abstract])) OR (("sheep milk"[Title/Abstract] OR "ewe milk"[Title/Abstract] OR "ovine milk"[Title/Abstract] OR "sheep yogurt"[Title/Abstract] OR "sheep yoghurt"[Title/Abstract] OR "ovine yogurt"[Title/Abstract] OR "ovine yoghurt"[Title/Abstract] OR "ewe yogurt"[Title/Abstract] OR "ewe yoghurt"[Title/Abstract]) AND (ferment*[Title/Abstract] OR digestion[Title/Abstract] OR "food matrix"[Title/Abstract] OR peptide*[Title/Abstract] OR mineral*[Title/Abstract] OR "medium-chain fatty acid*"[Title/Abstract] OR microbiota[Title/Abstract] OR "intestinal barrier"[Title/Abstract] OR "gut-liver axis"[Title/Abstract] OR "alcohol-associated liver disease"[Title/Abstract] OR "ethanol-induced liver injury"[Title/Abstract]))) AND ("2006/01/01"[Date - Publication] : "2026/02/12"[Date - Publication])
Web of Science Core Collection	12 February 2026; publication-date filter: 1 January 2006–12 February 2026	TS=((("alcohol-associated liver disease" OR "alcohol-related liver disease" OR "alcoholic liver disease" OR "alcoholic hepatitis" OR "ethanol-induced liver injury" OR "alcohol-induced liver injury") AND ("gut-liver axis" OR "intestinal barrier" OR "intestinal permeability" OR "tight junction*" OR mucus OR dysbiosis OR microbiota OR microbiome OR

		mycobiome OR Candida OR "microbial translocation" OR "fungal translocation" OR lipopolysaccharide OR endotoxemia OR "bile acid*" OR FXR OR TGR5 OR FGF15 OR FGF19 OR tryptophan OR indole* OR "aryl hydrocarbon receptor" OR AhR OR zinc OR malnutrition OR sarcopenia OR yogurt OR yoghurt OR "fermented dairy" OR "fermented milk" OR kefir OR probiotic* OR postbiotic* OR "lactic acid bacteria")) OR (("sheep milk" OR "ewe milk" OR "ovine milk" OR "sheep yogurt" OR "sheep yoghurt" OR "ovine yogurt" OR "ovine yoghurt" OR "ewe yogurt" OR "ewe yoghurt") AND (ferment* OR digestion OR "food matrix" OR peptide* OR mineral* OR "medium-chain fatty acid*" OR microbiota OR "intestinal barrier" OR "gut-liver axis" OR "alcohol-associated liver disease" OR "ethanol-induced liver injury"))))
Scopus	12 February 2026; publication-date filter: 1 January 2006–12 February 2026	TITLE-ABS-KEY(((("alcohol-associated liver disease" OR "alcohol-related liver disease" OR "alcoholic liver disease" OR "alcoholic hepatitis" OR "ethanol-induced liver injury" OR "alcohol-induced liver injury") AND ("gut-liver axis" OR "intestinal barrier" OR "intestinal permeability" OR "tight junction*" OR mucus OR dysbiosis OR microbiota OR microbiome OR mycobiome OR Candida OR "microbial translocation" OR "fungal translocation" OR lipopolysaccharide OR endotoxemia OR "bile acid*" OR FXR OR TGR5 OR FGF15 OR FGF19 OR tryptophan OR indole* OR "aryl hydrocarbon receptor" OR AhR OR zinc OR malnutrition OR sarcopenia OR yogurt OR yoghurt OR "fermented dairy" OR "fermented milk" OR kefir OR probiotic* OR postbiotic* OR "lactic acid bacteria")) OR (("sheep milk" OR "ewe milk" OR "ovine milk" OR "sheep yogurt" OR "sheep yoghurt" OR "ovine yogurt" OR "ovine yoghurt" OR "ewe yogurt" OR "ewe yoghurt") AND (ferment* OR digestion OR "food matrix" OR peptide* OR mineral* OR "medium-chain fatty acid*" OR microbiota OR "intestinal barrier" OR "gut-liver axis" OR "alcohol-associated liver disease" OR "ethanol-induced liver injury"))))
Google Scholar	12 February 2026; custom date range: 2006–2026	Because Google Scholar limits query length, run the following targeted searches separately: (1) "alcohol-associated liver disease" ("gut-liver axis" OR "intestinal barrier" OR microbiome OR mycobiome OR "microbial translocation"); (2) ("alcohol-associated liver disease" OR "ethanol-induced liver injury") (yogurt OR "fermented milk" OR kefir OR probiotic OR postbiotic OR "lactic acid bacteria"); (3) ("sheep yogurt" OR "ovine yogurt" OR "sheep milk" OR "ovine milk") (fermentation OR digestion OR "food matrix" OR peptide OR microbiota OR "intestinal barrier" OR "alcohol-associated liver disease"); (4) ("alcohol-associated liver disease" OR "alcoholic liver disease") ("bile acid" OR FXR OR TGR5 OR tryptophan OR indole OR "aryl hydrocarbon receptor" OR AhR OR zinc OR malnutrition). Reference-list screening and backward/forward citation tracking were also undertaken.

* The asterisk indicates truncation where supported by the database. Database-specific syntax and date filters were adapted to the relevant platform. Google Scholar searches were conducted as supplementary targeted searches because of query-length and reproducibility limitations.

Supplementary Table S2. Representative evidence streams informing the sheep-yogurt matrix research framework.

Evidence stream	Role in this review	Representative references
ALD clinical scope, nutrition, and management context	Defines ALD context and nutritional vulnerability without implying sheep-yogurt therapy	[8–10,22–26]
Gut–liver axis and bacterial dysbiosis	Supports ALD as gut–liver axis disorder and motivates microbiome readouts	[11,12,28–30]
Barrier dysfunction and microbial translocation	Supports permeability, tight-junction, mucus, and translocation endpoints	[13,33,97]
Mycobiome and fungal exposure	Supports Candida, β-glucan, and fungal immune readouts	[14–16,34–36]
Bile acid signaling	Supports FXR, TGR5, FGF15/19, bile acid profiling, and BSH-related hypotheses	[17–19,37–41,77,108]
AhR–tryptophan and IL-22 defense	Supports AhR, indole, IL-22, and REG3-related readouts	[20,21,42–48]
Probiotics, fermented milk, and postbiotics in ALD models	Supports microbial-intervention comparators but not sheep-yogurt efficacy	[50,51,65,75,76,78–82,100]
Fermented food and food-matrix mechanisms	Supports matrix-level framing and digestion/carrier effects	[6,7,27,56,64,67–69,107]
Sheep milk, sheep yogurt, and ovine-derived components	Supports ovine-substrate comparison and sheep-dairy intestinal context	[1–3,52–55,63]
Goat milk and caprine fermented dairy comparators	Supports goat yogurt and caprine dairy as non-equivalent comparators	[5,57–62]
Non-ALD epithelial barrier and postbiotic models	Supports barrier assay selection without implying sheep-yogurt ALD efficacy	[66,70–74,106]
Organoid, Liver-Chip, and gut–liver platforms	Supports model-system selection for future testing	[87–96]
Additional fermented milk and colitis models	Supports indirect intestinal-inflammation context	[101–105]
Translational uncertainty, biomarkers, and stratification	Supports safety-aware and stratified research agenda	[84–86,98,99]

References are presented as numerical citations in square brackets and correspond to the reference list.
